# Clinical validation of the novel HDAC6 radiotracer [^18^F]EKZ-001 in the human brain

**DOI:** 10.1007/s00259-020-04891-y

**Published:** 2020-07-08

**Authors:** Michel Koole, Donatienne Van Weehaeghe, Kim Serdons, Marissa Herbots, Christopher Cawthorne, Sofie Celen, Frederick A. Schroeder, Jacob M. Hooker, Guy Bormans, Jan de Hoon, Janice E. Kranz, Koen Van Laere, Tonya M. Gilbert

**Affiliations:** 1 Nuclear Medicine and Molecular Imaging, Department of Imaging and Pathology, KU Leuven, 3000 Leuven, Belgium; 2 Nuclear Medicine and Molecular Imaging, University Hospitals Leuven, 3000 Leuven, Belgium; 3 Center for Clinical Pharmacology, University Hospitals Leuven, 3000 Leuven, Belgium; 4 Laboratory for Radiopharmaceutical Research, KU Leuven, 3000 Leuven, Belgium; 5 Eikonizo Therapeutics, Inc., LabCentral, 700 Main Street North, Cambridge, MA 02139 USA; 6 Athinoula A. Martinos Center for Biomedical Imaging, Department of Radiology, Massachusetts General Hospital, Harvard Medical School, Charlestown, MA 02129 USA

**Keywords:** HDAC6, Positron emission tomography, Neuroimaging, [^18^F]EKZ-001, [^18^F]Bavarostat, First-in-human

## Abstract

**Purpose:**

Histone deacetylase 6 (HDAC6) is a cytoplasmic enzyme that modulates intracellular transport and protein quality control. Inhibition of HDAC6 deacetylase activity has shown beneficial effects in disease models, including Alzheimer’s disease and amyotrophic lateral sclerosis. This first-in-human positron emission tomography (PET) study evaluated the brain binding of [^18^F]EKZ-001 ([^18^F]Bavarostat), a radiotracer selective for HDAC6, in healthy adult subjects.

**Methods:**

Biodistribution and radiation dosimetry studies were performed in four healthy subjects (2M/2F, 23.5 ± 2.4 years) using sequential whole-body PET/CT. The most appropriate kinetic model to quantify brain uptake was determined in 12 healthy subjects (6M/6F, 57.6 ± 3.7 years) from 120-min dynamic PET/MR scans using a radiometabolite-corrected arterial plasma input function. Four subjects underwent retest scans (2M/2F, 57.3 ± 5.6 years) with a 1-day interscan interval to determine test-retest variability (TRV). Regional volume of distribution (*V*_*T*_) was calculated using one-tissue and two-tissue compartment models (1-2TCM) and Logan graphical analysis (LGA), with time-stability assessed. *V*_*T*_ differences between males and females were evaluated using volume of interest and whole-brain voxel-wise approaches.

**Results:**

The effective dose was 39.1 ± 7.0 μSv/MBq. Based on the Akaike information criterion, 2TCM was the preferred model compared to 1TCM. Regional LGA *V*_*T*_ were in agreement with 2TCM *V*_*T*_, however demonstrated a lower absolute TRV of 7.7 ± 4.9%. Regional *V*_*T*_ values were relatively homogeneous with highest values in the hippocampus and entorhinal cortex. Reduction of acquisition time was achieved with a 0 to 60-min scan followed by a 90 to 120-min scan. Males demonstrated significantly higher *V*_*T*_ than females in the majority of cortical and subcortical brain regions. No relevant radiotracer related adverse events were reported.

**Conclusion:**

[^18^F]EKZ-001 is safe and appropriate for quantifying HDAC6 expression in the human brain with Logan graphical analysis as the preferred quantitative approach. Males showed higher HDAC6 expression across the brain compared to females.

## Introduction

Histone deacetylase 6 (HDAC6) is a cytoplasmic enzyme that contains two catalytic domains, a dynein motor binding domain and a ubiquitin-binding zinc finger domain (ZnF-UBP). HDAC6 belongs to the class IIb HDAC family and, unlike class I HDACs, does not deacetylate histones. Instead, HDAC6 removes acetyl groups from multiple substrates including tubulin [1, 2], tau [3, 4], HSP90 [5, 6], and cortactin [7], and thus influences diverse cellular processes such as intracellular transport [8], protein quality control [9], and cell migration [7]. HDAC6 also coordinates the clearance of protein aggregates through the ZnF-UBP [10–12]. Modulation of HDAC6 expression levels or enzymatic activity has shown beneficial effects in multiple cell and animal models of disease, including neurodegenerative disorders [13–23], psychiatric disorders [24, 25], peripheral neuropathies [26–33], and cancer [34, 35]. Therefore, quantifying the expression and distribution of HDAC6 in humans is of great importance to understand how HDAC6 may relate to disease pathogenesis, progression, and treatment response.

HDAC6, encoded on the X-chromosome, is expressed throughout the human body, with relatively high expression levels reported in brain [36, 37]. HDAC6 protein is detectable in postmortem human brain tissue across gray and white matter, in cortical, subcortical, and infratentorial regions [38, 39]. Localized alterations of HDAC6 protein levels in brain disorders were found in a limited number of ex vivo studies. For example, compared to healthy controls, HDAC6 was elevated in the temporal cortex of donor samples from patients with frontotemporal lobar degeneration with TDP-43 inclusions (FTD-TDP43) [40] and in the frontal cortex and hippocampus from patients with Alzheimer’s disease [41, 42], as well as in tumors from patients with glioblastoma [34, 35]. However, the methodological constraints of measuring HDAC6 expression in postmortem tissue have impeded the number of brain regions and disease states investigated and precluded longitudinal studies.

Positron emission tomography (PET) offers a non-invasive method to measure in vivo protein expression in the living brain. Previously, the less selective radiotracer [^11^C]Martinostat [43–46], with affinity for HDAC paralogs 1, 2, 3, and to a lesser extent 6, was successfully implemented in human neuroimaging studies [38, 47–49]. [^11^C]Martinostat PET showed lower relative HDAC expression in the dorsolateral prefrontal cortex of patients with schizophrenia or schizoaffective disorder compared to controls [47], whereas no significant differences between patients with amyotrophic lateral sclerosis (ALS) and controls were observed [49]. Nevertheless, it has been demonstrated in ALS that different HDAC paralogs might have conflicting roles [23, 50–53] and paralog-selective HDAC radiotracers are needed.

Recently, preclinical HDAC6-selective radiotracers have been developed [54–56], including the highly brain-penetrant [^18^F]Bavarostat (hereafter called [^18^F]EKZ-001) [55]. In biochemical assays EKZ-001 displayed low nanomolar potency (< 20 nM) for HDAC6 and > 100-fold selectivity for HDAC6 over class I HDACs [55, 57]. In human neural progenitor cells, EKZ-001 inhibited deacetylation of the HDAC6 substrate α-tubulin, but not deacetylation of the class I HDAC substrates (histone H3 on lysine 9 and histone H4 on lysine 12) [55]. In in vitro autoradiography competition assays, [^18^F]EKZ-001 binding was blocked by preclinical and clinical HDAC6 inhibitors, with compounds that have higher HDAC6 selectivity demonstrating higher blocking efficiency [55, 57]. In non-human primate PET studies, [^18^F]EKZ-001 demonstrated high specific binding [55], uptake was blocked by highly selective HDAC6 inhibitors in a dose-dependent manner [57], and binding kinetics were favorable for calculating volume of distribution (*V*_*T*_) estimates [57]. Finally, a fully automated current good manufacturing practice (cGMP) compliant production method for human use was developed to facilitate clinical translation of [^18^F]EKZ-001 PET [57]. Here, we performed a first-in-human [^18^F]EKZ-001 PET study to investigate biodistribution and dosimetry, as well as kinetic modeling in brain, short-term test-retest variability, inter-subject variability, and a clinically relevant PET imaging protocol by maximal reduction of scanning time.

## Materials and methods

### Study design

The primary objectives of this open-label phase 1 study were threefold (cohorts A, B, and C). In cohort A, we estimated biodistribution and whole-body dosimetry of [^18^F]EKZ-001 in healthy adult subjects. In cohort B, we measured [^18^F]EKZ-001 uptake in the brain of healthy adult subjects and modeled radiotracer kinetics with the appropriate radiometabolite-corrected arterial input function. An age range of 50–64 years was selected to facilitate future investigation of neurodegenerative disorders relevant to HDAC6. We then evaluated test-retest variability (TRV) of [^18^F]EKZ-001 uptake by comparing the quantification of two PET scans of the same subject, obtained 1 day apart. In cohort C, we further extended the dynamic datasets of cohort B to evaluate inter-subject variability of [^18^F]EKZ-001 uptake by comparing the quantification of PET scans between healthy adult subjects and examine potential sex differences. Additionally, we considered a reduction of the PET acquisition time by either shortening the scanning duration or splitting the scan into two sessions with an intermediate break (coffee-break protocol) to facilitate future translation to patient populations. The study was approved by the independent Ethics Committee of the University Hospitals of KU Leuven and was performed in accordance with the World Medical Association Declaration of Helsinki. Written informed consent was obtained from all volunteers prior to the study.

### Study subjects and eligibility

Subjects were recruited by using the healthy volunteer database of the Center for Clinical Pharmacology. Four healthy subjects (2M/2F, mean age 23.5 ± 2.4 years) in cohort A, four healthy subjects (2M/2F, mean age 57.3 ± 5.6 years) in cohort B, and twelve healthy subjects (6M/6F, mean age 57.6 ± 3.7 years, encompassing the subjects in cohort B) in cohort C were included. All subjects had a body mass index ≥ 18.5 kg/m^2^ and < 30.0 kg/m^2^ and were carefully screened. The main exclusion criteria were abnormal physical or neurological examination or paraclinical investigations (using standard laboratory tests and electrocardiograms), history of significant medical illnesses including major internal pathology or neurological and neuropsychiatric disorders, history of clinically relevant drug or food allergies, use of tobacco products (up to 5 years before screening), use of illicit drugs (as assessed by a urine drug test), and significant abnormalities on an anatomical magnetic resonance (MR) scan.

### [^18^F]EKZ-001 synthesis according to cGMP

[^18^F]EKZ-001 was synthesized starting from the phenolic precursor via innovative ruthenium-mediated radiofluorination chemistry [55] with a fully automated cGMP production method using a commercial radiofluorination module (Trasis All-In-One; Trasis, Liège, Belgium). The radiotracer was obtained with high radiochemical purity (> 98%) in a total synthesis time of 2 h with an average molar activity of 204 ± 175 GBq/μmol (*n* = 23, including the validation runs) at the end of the synthesis process. Detailed synthesis methods are described in Celen et al. [57].

### Subject dosing

All subjects fasted for at least 4 h prior to PET imaging. Subjects received an average dose of 142 ± 32 MBq (range [115 MBq - 201 MBq]) of [^18^F]EKZ-001 as a manual intravenous bolus injection under standardized injection conditions (supine, low ambient noise, dimly lit room) through a catheter in a peripheral vein. The average injected mass of [^18^F]EKZ-001 was 0.70 ± 0.73 μg (range [0.11–3.61 μg]) with an average molar activity of 115 ± 86 GBq/μmol (range [17–384 GBq/μmol]) at time of injection. In total, 16 healthy subjects (cohorts A, B, and C) were included. Demographic data and dose information are summarized for each study cohort in Table 1.

**Table 1:** Subject demographic data and dose information including net injected doses of [^18^F]EKZ-001 with corresponding injected mass, molar activity (MA) and plasma free fraction (PFF) when appropriate. Scans took place between 13 May 2019 and 4 September 2019 with time of injection between 12:30 and 15:30 PM

Subject code	Sex	Weight (kg)	Age (Y)	Scan	Activity (MBq)	Injected mass (ug)	MA (GBq/umol)	PFF (%)
Cohort A								
1	M	71	23	Scan 1	190	0.72	91.6	–
2	M	71	22	Scan 1	179	3.61	17.1	–
3	F	51	27	Scan 1	166	0.43	133	–
4	F	80	22	Scan 1	174	0.47	128	–
Cohort B								
5	M	74	61	Scan 1	194	0.89	75.5	–
	M	73	61	Scan 2	116	0.62	64.6	–
6	F	79	53	Scan 1	121	0.65	64.8	0.66
	F	79	53	Scan 2	127	0.62	70.6	0.70
7	M	75	52	Scan 1	116	0.51	78.7	0.47
	M	75	52	Scan 2	120	0.36	114	2.12
8	F	51	63	Scan 1	116	1.19	33.7	0.61
	F	51	63	Scan 2	126	0.90	48.9	–
Cohort C								
9	M	73	58	Scan 1	182	0.29	217	–
10	M	81	58	Scan 1	201	0.79	87.8	–
11	F	77	53	Scan 1	120	0.43	95.4	1.18
12	F	69	57	Scan 1	122	0.22	196	1.33
13	F	79	57	Scan 1	116	0.57	71.1	1.10
14	F	55	57	Scan 1	116	0.11	384	1.47
15	M	83	63	Scan 1	132	0.19	245	1.01
16	M	77	59	Scan 1	116	0.46	87.9	1.26

### Whole-body dosimetry (cohort A)

Four healthy subjects (2M/2F, see Table 1, cohort A) underwent 13 consecutive PET-CT scans, covering the whole-body (WB) radiotracer distribution for approximately 300 min after radiotracer administration. WB PET-CT scans were acquired from the midfemoral position to the head in three segments (11 sequential WB scans from radiotracer injection to 90 min, a 12th scan at 150 min and a 13th scan at 270 min after radiotracer injection, respectively).

WB PET-CT scans were performed on a 40-slice PET-CT camera (Biograph Truepoint; Siemens, Ehrlangen, Germany). During positioning, the head was restrained using a vacuum cushion and the body was strapped to the bed in order to minimize movement during the scan. Bed height was kept constant between scanning segments. All data were corrected for random coincident detection, scatter, and attenuation. Data were reconstructed using a three-dimensional (3D) ordered-subset expectation-maximization (OSEM) iterative reconstruction with 5 iterations and 8 subsets, and postsmoothing with a 3D Gaussian kernel (full width half maximum (FWHM) of 6 mm). For the attenuation correction, a low-dose (80 kV tube potential, 11 effective mAs) WB CT scan was acquired at the beginning of each PET acquisition segment to generate a CT-based attenuation map.

Volumes of interest (VOIs) were constructed in 3D on the PET emission data to include all organ activity from source organs with a significant and visually assessable amount of radiotracer uptake (brain, gallbladder wall, small intestine, upper large intestine, lower large intestine, heart wall, kidneys, lungs, liver, red marrow, spleen, testes when appropriate, thyroid, urinary bladder, and the total body from which the remainder of the body is calculated). VOI positions were verified on the corresponding CT images and visually inspected for possible movement between sequential scans within and between segments. Residual errors were corrected by manually redefining VOIs (necessary in all subjects for the gallbladder). Bone marrow radiotracer uptake was considered as an estimate for red marrow. The International Commission on Radiological Protection (ICRP 30) gastrointestinal (GI) model (ICRP 1979) was used to determine the normalized cumulated activities (NCA) for the organs involved in the GI tract with the fraction of injected activity entering the small intestine set equal to the decay-corrected plateau fraction of injected activity encompassed by a large abdominal VOI. To account for the timing differences between bed positions, the acquisition time of the bed position corresponding to the axial midposition of the source organ under consideration was used to calculate the individual acquisition time for each source organ. In this way, time-activity curves (TAC) were extracted for each of the abovementioned source organs and NCA values were calculated by normalizing the area under curve of the TAC of each source organ to the total injected activity, corrected for the residual activity in the syringe and injection line. For this purpose, a dual exponential ${\mathrm{A}}_1\exp \left(-\frac{0.69\mathrm{t}}{\uptau_{e,1}}\right)+{\mathrm{A}}_2\exp \left(-\frac{0.69\mathrm{t}}{\uptau_{e,2}}\right)$ was fit to the TACs of heart wall, kidneys, lungs, spleen, and thyroid while the function ${\mathrm{A}}_1\left(1-\exp \left(-\frac{0.69\mathrm{t}}{\uptau_{u,1}}\right)\right)\exp \left(-\frac{0.69\mathrm{t}}{\uptau_{e,1}}\right)+{\mathrm{A}}_2\exp \left(-\frac{0.69\mathrm{t}}{\uptau_{e,2}}\right)$ was fit to the TACs of brain, liver, and red marrow and the function ${\mathrm{A}}_1\left(1-\exp \left(-\frac{0.69\mathrm{t}}{\uptau_{u,1}}\right)\right)+{\mathrm{A}}_2\exp \left(-\frac{0.69\mathrm{t}}{\uptau_{e,2}}\right)$ was fit to the TAC of the remainder, respectively. In these expressions, A_i_ (i = 1, 2) represents a scaling parameter while τ_*e*, *i*_ (i = 1, 2) represents an excretion time parameter and τ_*u*, 1_ represents an uptake time parameter. These curve models were fit to the data by least-squares constraint minimization. For the uptake in the gallbladder, testes and urinary bladder, no curve model was found to give a satisfactory fit and therefore only values based on the trapezoid rule were retained. The NCA values for the upper large intestine, lower large intestine, and small intestine were calculated using the ICRP 30 GI model [58] as incorporated in OLINDA v1.1 [59] using the fraction entering the small intestine as input.

Based on the NCA values, absorbed doses were calculated using OLINDA v1.1 [59], according to current ICRP 60 definitions and using the medical internal radiation dose (MIRD) scheme of a 73.7-kg male adult for the male subjects and a 56.9-kg female adult for the female subjects. The effective dose (ED) was calculated from the individual organs doses based on predefined organ weighting factors, as specified by ICRP 60 in 1991 [60].

### Radiotracer kinetic modeling, test-retest and inter-subject variability in brain (cohorts B–C)

Twelve healthy subjects (6M/6F, see Table 1, cohorts B–C) completed a dynamic time-of-flight (TOF) PET-MR brain scan combined with arterial blood sampling and radiometabolite analysis (General Electric Signa, Milwaukee, USA). Dynamic PET imaging was performed in list mode for 120 min, re-binned in 31 frames (6 × 15 s, 3 × 30 s, 3 × 1 min, 2 × 1.5 min, 2 × 3 min, 9 × 5 min, 6 × 10 min) and corrected for deadtime, random coincident detection, and scatter. A zero-echo time (ZTE)-based approach, which was quantitatively validated to PET-CT [61], was used to generate a MR-based attenuation map to correct for attenuation on an individual subject basis [62]. Reconstruction of all frames was performed using OSEM with 28 subsets and 6 iterations and included TOF information, resolution modeling, and postsmoothing with a 3D Gaussian kernel (FWHM of 4 mm). Dynamic PET data were checked and corrected for motion using a frame by frame approach and performing rigid co-registration of each frame with the average of the first 12 frames representing the first 9 min of the PET acquisition.

Before the start of the PET scan, an arterial blood sample was taken to determine the free fraction of [^18^F]EKZ-001 in plasma (PFF) using an ultrafiltration method [43]. During the dynamic PET scan, manual arterial blood sampling was performed from time of injection to 100 min postinjection, with time intervals gradually increasing towards the end. A three-exponential model curve was fitted to the blood and plasma activity values while a mono-exponential function was fitted to the plasma radiometabolite data.

Simultaneous with the PET acquisition, a 3D volumetric T1-weighted brain volume imaging (BRAVO – sagittal direction; TE: 3.2 ms; TR: 8.5 ms; TI: 450 ms; Flip Angle: 12; Receiver Bandwidth: 31.2; NEX: 1; voxel size: 1x1x1 mm) sequence and 3D T2-weigthed CUBE fluid attenuated inversion recovery (FLAIR - sagittal direction; TE: 137 ms; #echoes: 1; echo train length: 190; TR: 8500 ms; TI: 50 ms; Receiver Bandwidth: 31.25; NEX: 1; voxel size: 1.2 × 1.3 × 1.4 mm) sequence were acquired. A SPM-based multichannel segmentation using both the 3D T1 BRAVO and interpolated T2 CUBE FLAIR was performed using standard settings (SPM12, Welcome Trust Centre for Neuroimaging, University College, London, UK) to determine subject specific tissue probability maps for gray matter, white matter and cerebrospinal fluid.

Subject-specific regional parcellation of the PET data was performed by rigid alignment of the motion corrected dynamic PET data with the 3D T1 BRAVO dataset and subsequently non-linear spatial normalization to Montreal Neurological Institute (MNI) space using the 3D T1 BRAVO in PNEURO v3.9 (PMOD technologies, Zurich, Switzerland). Regional TACs were extracted to project the predefined brain regions of a simplified Hammers atlas. The orbitofrontal, frontal, motor, entorhinal, temporal, parietal, and occipital cortex were delineated as well as the insula, cerebellum, posterior and anterior cingulate, caudate nucleus, putamen, thalamus, hippocampus, and brainstem. Left and right VOIs were grouped. Brain VOIs were restricted to gray matter by taking advantage of the patient specific gray matter probability map resulting from the multichannel MR segmentation in SPM12. For this purpose, a fixed threshold of 0.3 was applied to the individual gray matter probability maps to include only voxels in a brain VOI with a high probability of belonging to gray matter. This procedure was used for all brain VOIs except for the brain stem and subcortical brain structures such as caudate nucleus, putamen, and thalamus.

Regional total distribution volume estimates (*V*_*T*_) were determined by applying 1- and 2-tissue compartment models (1TCM–2TCM) with fixed blood volume of 5% and using the composite cortical TAC to estimate potential small time shifts between the PET TAC and arterial blood/plasma input functions. The Akaike information criterion (AIC) was used to select the most appropriate compartment model for *V*_*T*_ estimation. Next, *V*_*T*_ values determined with the most appropriate compartment model were compared with *V*_*T*_ estimates using a non-compartment Logan graphical analysis (LGA) [63] to validate LGA as a fast and straightforward method for estimating voxel-wise *V*_*T*_ maps and regional *V*_*T*_ values.

In cohort B, four healthy subjects (2M/2F) received a second retest PET scan with an interscan interval of 1 day. For the retest scan, the same PET-MR protocol as well as the same processing steps were used as for the first PET scan. TRV and reliability of *V*_*T*_ values were determined for the most appropriate compartment model and for LGA. For this purpose, TRV was assessed for each brain VOI as $2\times \left({V}_T^{test}-{V}_T^{retest}\right)/\left({V}_T^{test}+{V}_T^{retest}\right)$ and absolute TRV (aTRV) as $2\times \mid {V}_T^{test}-{V}_T^{retest}\mid /\left({V}_T^{test}+{V}_T^{retest}\right)$ both averaged over the 4 test-retest datasets. Reliability was evaluated using the intra-class correlation coefficient (ICC), calculated as (BSMSS-WSMSS)/(BSMSS+WSMSS) with BSMSS and WSMSS the mean sum of squares (MSS) between subjects (BS) and within subjects (WS), respectively. Therefore, ICC evaluates WS variability relative to BS variability and ranges from − 1 to 1, with values closer to 1 indicating better reliability.

In cohorts B–C, inter-subject variability was assessed by the coefficient of variation (%COV) of the *V*_*T*_ values determined for the most appropriate compartment model and for LGA, for each brain region across the 12 healthy subjects (6M/6F) and for male and female subjects separately, using only the first PET scan for subjects with two scans. Regional LGA *V*_*T*_ values were compared between male and female subjects using a two-way analysis of variance (ANOVA). Additionally, a whole-brain voxel-wise analysis was performed in SPM 12 after spatial normalization to MNI space and isotropic Gaussian smoothing of 6 mm. The threshold was set at *p*_height uncorr_ < 0.001 and *p*_cluster FWE-corr_ < 0.05.

Time stability of the regional LGA *V*_*T *_values was evaluated by either shortening the scanning time or splitting the scan into two sessions with an intermediate break (coffee-break protocol). For the first approach, the acquisition time of one PET scan session, starting at radiotracer injection, was shortened down to 60 min and regional *V*_*T *_values were compared with a 120-min dynamic PET scan starting at radiotracer injection. For the second approach, the PET acquisition was split into two dynamic scanning sessions, one 60-min session starting at radiotracer injection, and one 30-min session starting at 90 min after radiotracer injection. Regional *V*_*T*_ values estimated with this coffee-break protocol were again compared with a 120-min dynamic PET scan starting at radiotracer injection. For both scenarios, time stability of regional *V*_*T*_ was evaluated in terms of TRV for the test-retest datasets (4 subjects, 2M/2F, cohort B) and bias compared to a 120-min dynamic scan, defined as the percentage relative difference between *V*_*T*_ values (12 subjects, 6M/6F, cohorts B–C, only the first scan was considered for subjects with two scans).

## Results

### Biodistribution and whole-body dosimetry

**Fig. 1:**
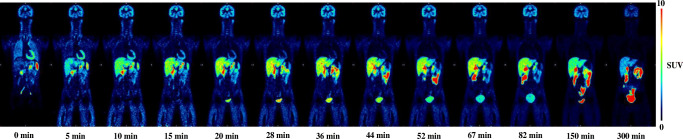
Whole-body time-activity distribution of [^18^F]EKZ-001 in subject number 1 (male, Table 1, cohort A), with representative coronal slices. PET image intensities are expressed as standard uptake value (SUV) and are visualized relative to the maximum color table values as indicated in the scale bar, to account for physical radiotracer decay. The times (min) indicate the start of the whole-body scan relative to radiotracer injection

A representative coronal series of [^18^F]EKZ-001 emission scans over time (subject 1 from Table 1, cohort A) is shown in Fig. 1. Whole-body biodistribution and routes of excretion of the radiotracer and its radiometabolites are visualized, with sustained uptake in the brain, heart, and skeletal muscle, and a mainly hepatobiliary mechanism of excretion observed with only limited renal clearance. Calculated NCA for all source organs with activity above background and the individual organ doses for all subjects (*n* = 4 subjects, 2M/2F, cohort A) with mean value, standard deviation, and range values are shown in Table 2 and Table 3, respectively. The fraction of injected activity entering the small intestine, determined as the decay-corrected plateau fraction of injected activity encompassed by the intestinal VOI, was 63%, 49%, 67%, and 68%, for each subject, respectively, and used as input for the ICRP 30 GI model (ICRP 1979) to determine the NCA for the organs involved in the GI tract. The upper large intestine wall showed the highest organ dose of 270 μGy/MBq with a maximal dose estimated to about 51 mGy, followed by the gallbladder (245 μGy/MBq) and the small intestine (244 μGy/MBq). The average ED was 39.1 ± 7.0 μSv/MBq, which is at the higher end of the typical range of 15–30 μSv/MBq for ^18^F-labeled radiopharmaceuticals [64]. Therefore, subjects in cohort A received an average effective dose of 6.9 ± 0.4 mSv for the PET scan.

**Table 2:** Normalized cumulated activities (NCA) of [^18^F]EKZ-001 for 13 source organs and the remainder with descriptive statistics given as mean ± SD [range] (*n* = 4 subjects, 2M/2F, cohort A). Additionally, testes were considered as source organs for the male subjects

Organ	NCA (MBq-hr/MBq)
Brain	0.169 ± 0.023 [0.151, 0.201]
Gallbladder wall	0.128 ± 0.030 [0.085, 0.152]
Lower large intestine wall	0.099 ± 0.014 [0.078, 0.109]
Small intestine	0.982 ± 0.139 [0.779, 1.081]
Upper large intestine wall	0.539 ± 0.077 [0.428, 0.593]
Heart wall	0.008 ± 0.004 [0.003, 0.012]
Kidneys	0.048 ± 0.016 [0.031, 0.064]
Liver	0.253 ± 0.088 [0.168, 0.376]
Lungs	0.036 ± 0.008 [0.028, 0.045]
Red marrow	0.033 ± 0.006 [0.027, 0.040]
Spleen	0.009 ± 0.002 [0.008, 0.011]
Testes	0.002 ± 0.001 [0.001, 0.002]
Thyroid	0.001 ± 0.000 [0.001, 0.001]
Urinary bladder wall	0.071 ± 0.030 [0.033, 0.105]
Remainder/total body	0.703 ± 0.206 [0.456, 0.899]

**Table 3:** Radiation-absorbed dose estimates of [^18^F]EKZ-001 for all 25 organs using the ICRP 30 gastro-intestinal (GI) tract model and the medical internal radiation dose (MIRD) scheme of a 73.7-kg male adult and a 56.9-kg female adult for the male and female subjects, respectively, using OLINDA v1.1 with descriptive statistics given as mean ± SD [range] (*n* = 4 subjects, 2M/2F, cohort A)

Organ	Organ doses (μGy/MBq)
Adrenals	13.9 ± 1.9 [12.4, 16.7]
Brain	31.0 ± 3.7 [26.3, 34.5]
Breasts	5.0 ± 0.2 [4.8, 5.2]
Gallbladder wall	245 ± 50 [186.0, 308.0]
Lower large intestine wall	87.1 ± 15.8 [65.9, 100.0]
Small intestine	244 ± 54 [176.0, 288.0]
Stomach wall	18.1 ± 2.6 [14.9, 20.6]
Upper large intestine wall	270 ± 53 [201.0, 312.0]
Heart wall	11.1 ± 2.0 [8.5, 12.7]
Kidneys	45.9 ± 11.7 [33.2, 60.7]
Liver	47.8 ± 17.5 [38.9, 74.0]
Lungs	11.9 ± 0.7 [10.9, 12.6]
Muscle	10.7 ± 1.0 [9.3, 11.4]
Ovaries	46.5 ± 9.8 [34.1, 54.7]
Pancreas	17.4 ± 2.1 [15.4, 20.3]
Red marrow	16.0 ± 1.0 [14.5, 16.7]
Osteogenic cells	12.7 ± 0.6 [12.0, 13.4]
Skin	5.7 ± 0.3 [5.4, 5.9]
Spleen	19.5 ± 1.0 [18.6, 20.8]
Testes	12.2 ± 3.8 [9.5, 14.9]
Thymus	5.5 ± 0.4 [5.1, 5.9]
Thyroid	9.5 ± 0.7 [8.8, 10.5]
Urinary bladder wall	54.7 ± 18.8 [27.3, 69.4]
Uterus	36.6 ± 7.4 [26.8, 42.5]
Remainder/total body	15.6 ± 1.9 [13.2, 17.6]

### Radiotracer kinetic modeling, test-retest, and inter-subject variability in brain

**Fig. 2:**
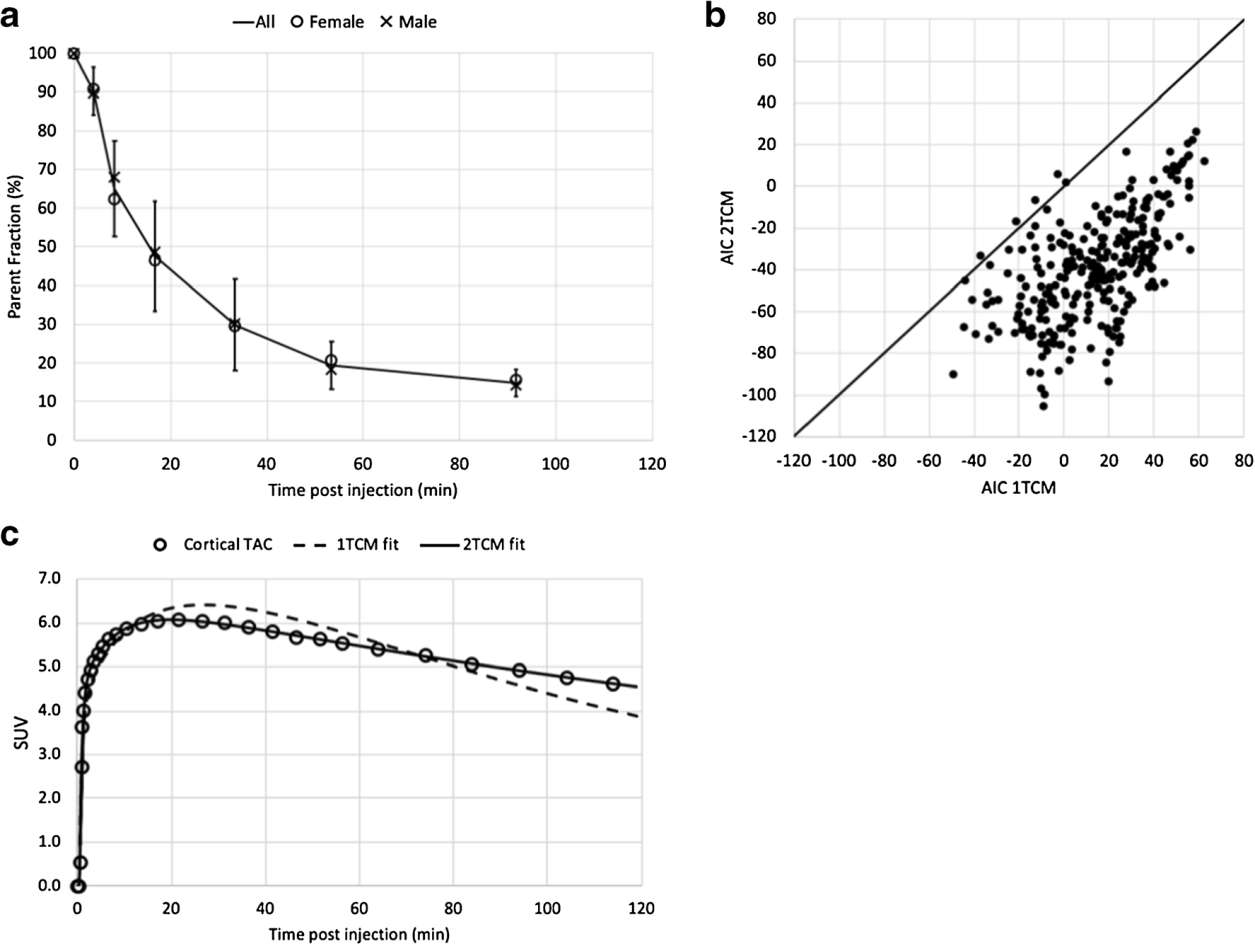
Metabolization rate of [^18^F]EKZ-001 for all subjects from cohorts B-C (mean with error bars ± SD) with average data points for males and females separately (**a**), one-tissue compartment model (1TCM) and two-tissue compartment model (2TCM) Akaike information criterion (AIC) values for model fitting to time activity curves (TACs) of different brain regions for dynamic [^18^F]EKZ-001 PET scans (*n* = 16 scans, 8M/8F inclusive of 2M/2F with two scans each, cohorts B–C) with a 120-min acquisition time (**b**) and representative 1TCM and 2TCM fitting results (subject 5, scan 1, see Table 1, cohort B) for the composite cortical TAC (**c**)

**Fig. 3:**
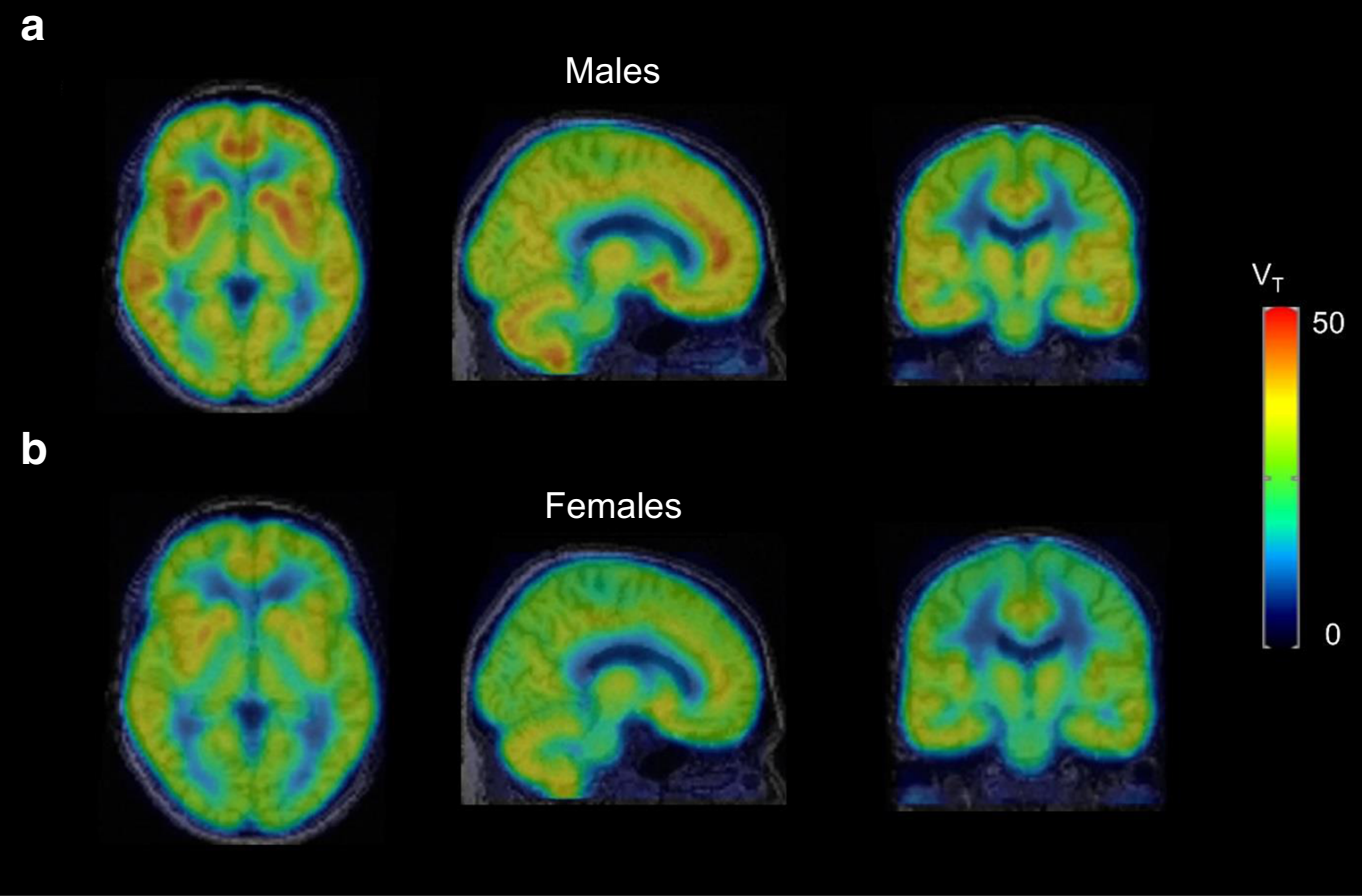
Average parametric Logan graphical analysis (LGA) *V*_*T*_ datasets for a 120-min [^18^F]EKZ-001 PET scan (*n* = 6 subjects per group, cohorts B–C, only the first scan was considered for subjects with 2 scans) for males (**a**) and females (**b**)

The PFF of [^18^F]EKZ-001 was determined for 9 subjects (3M/6F, see Table 1). PFF was 1.0 ± 0.4%, considering only the value from the first PET scan for the subject with two scans. Radiometabolite rates were similar between males and females (6M/6F) as shown in Fig. 2a. Regional TACs of the dynamic PET brain scans (Table 1 cohorts B–C: *n* = 16 scans, 8M/8F inclusive of 2M/2F with two scans each) were fit with 1TCM and 2TCM to identify the most appropriate kinetic model for measuring [^18^F]EKZ-001 uptake. The 2TCM AIC values were lower than 1TCM AIC values in 269 out of the 272 cases (98.9%) as shown in Fig. 2b. The 1TCM proved to be a more suitable model than 2TCM for only the caudate nucleus in 3 out of the 16 scans (18.8%). Representative 1TCM and 2TCM fits for a composite cortical TAC are shown in Fig. 2c. Together, these data clearly demonstrate that 2TCM is the preferred model over 1TCM for quantifying [^18^F]EKZ-001 uptake in brain. Therefore, we only report 2TCM and LGA *V*_*T*_ (using t* (starting time) = 40 min postinjection) values, which are presented in Table 4 (*n* = 12 subjects, 6M/6F, cohorts B-C). Mean *V*_*T*_ values were 39.4 ± 8.7 and 39.0 ± 7.6 for 2TCM and LGA, respectively, with high agreement between regional *V*_*T *_values as assessed by Bland-Altman analysis. The highest *V*_*T*_ was observed in the hippocampus and entorhinal cortex (mean LGA *V*_*T*_ of 50.4 and 46.4, respectively) and the lowest *V*_*T*_ was observed in the brainstem (mean LGA *V*_*T*_ of 26.9). No significant correlation was found between PFF and *V*_*T*_ (LGA *V*_*T*_ from cortical composite, Spearman’s rho, *p* = 0.78). Mean parametric LGA *V*_*T*_ maps generated from dynamic 120 min [^18^F]EKZ-001 PET scans in males or females (*n* = 6 per group) are shown in Fig. 3a, b.

**Table 4:** Two-tissue compartment model (2TCM) and Logan graphical analysis (LGA) *V*_*T*_ using an acquisition time of 120 min (*n* = 12 subjects, 6M/6F, cohorts B-C, only the first scan was considered for subjects with 2 scans) combined with a Bland Altman analysis comparing 2TCM and LGA *V*_*T*_ values. In addition, LGA *V*_*T*_ using an acquisition time of 120 min are reported separately for male and female subjects (6 M/6F, cohorts B-C). Descriptive statistics are given as mean ± SD (%COV) [range] while the Bland-Altman analysis is summarized as bias ± SD [95% limits of agreement]. Brain regions with significant differences between male and female LGA *V*_*T*_ (two-way ANOVA using Bonferroni’s multiple comparisons tests) are marked with **** (*p* ≤ 0.0001), ** (*p* ≤ 0.01), and * (*p* < 0.05)

	2TCM *V*_*T*_	LGA *V*_*T*_	Bland-Altman (2TCM vs LGA *V*_*T*_)	LGA *V*_*T*_ (6M)	LGA *V*_*T*_ (6F)
Composite cortical	37.2 ± 5.9 (15.9)[29.2, 47.5]	36.9 ± 4.8 (13.0)[29.0, 44.2]	− 0.4 ± 4.8[− 9.8, 9.0]	40.4 ± 3.0 (7.5)[36.1, 44.2]	33.4 ± 3.6 (10.6)[29.0, 38.9]
Orbitofrontal cortex	37.6 ± 6.1 (16.2)[29.9, 50.9]	36.6 ± 4.5 (12.4)[28.3, 43.6]	− 2.2 ± 6.7[− 15.4, 11.0]	39.6 ± 3.5 (8.8)[35.6, 43.6]	33.7 ± 3.5 (10.4)[28.3, 37.9]
Frontal cortex	36.2 ± 6.2 (17.0)[28.3, 47.2]	36.3 ± 5.0 (13.8)[28.0, 44.0]	0.6 ± 4.7[− 8.5, 9.8]	39.7 ± 3.5 (8.9)[34.5, 44.0]	32.9 ± 3.8 (11.7)[28.0, 38.6]
Motor cortex	33.1 ± 5.9 (17.8)[25.0, 44.9]	32.7 ± 4.6 (14.0)[25.0, 39.8]	− 0.8 ± 5.6[− 11.7, 10.1]	35.9 ± 3.4 (9.4)[31.3, 39.8]	29.5 ± 3.2 (11.0)[25.0, 34.4]
Temporal cortex*	42.5 ± 6.3 (14.9)[33.4, 53.8]	41.7 ± 5.2 (12.5)[32.9, 48.6]	− 1.8 ± 5.4[− 12.3, 8.8]	45.3 ± 2.9 (6.4)[41.0, 48.6]	38.0 ± 4.4 (11.5)[32.9, 44.6]
Parietal cortex	35.4 ± 5.9 (16.6)[27.9, 45.2]	35.4 ± 4.9 (13.8)[27.6, 43.3]	− 0.6 ± 4.4[− 8.0, 9.1]	38.9 ± 3.6 (9.2)[34.9, 43.3]	31.9 ± 3.2 (10.2)[27.6, 37.1]
Occipital cortex	34.6 ± 5.7 (16.5)[28.0, 46.9]	33.9 ± 4.0 (11.8)[28.2, 40.1]	− 1.5 ± 6.2[− 13.7, 10.7]	36.7 ± 3.2 (8.8)[32.1, 40.1]	31.0 ± 2.3 (7.4)[28.2, 33.9]
Insula*	43.8 ± 7.0 (16.0)[33.6, 60.0]	43.8 ± 5.8 (13.2)[33.7, 51.7]	0.5 ± 5.5[− 10.4, 11.3]	47.6 ± 3.9 (8.2)[41.8, 51.7]	40.1 ± 4.9 (12.3)[33.7, 47.5]
Anterior cingulate**	43.9 ± 7.2 (16.3)[32.9, 57.4]	44.1 ± 6.4 (14.5)[32.2, 52.9]	0.6 ± 5.1[− 9.4, 10.6]	48.7 ± 3.5 (7.1)[43.3, 52.9]	39.5 ± 5.2 (13.1)[32.2, 46.5]
Posterior cingulate	38.7 ± 6.1 (15.7)[30.9, 52.3]	39.2 ± 4.8 (12.2)[31.0, 46.8]	1.7 ± 5.0[− 8.1, 11.5]	42.4 ± 3.5 (8.3)[37.7, 46.8]	36.0 ± 3.7 (10.2)[31.0, 41.1]
Cerebellum	44.1 ± 7.6 (17.2)[34.1, 60.8]	43.5 ± 5.9 (13.6)[35.2, 53.1]	− 1.1 ± 5.9[− 12.7, 10.5]	46.5 ± 5.7 (12.2)[37.7, 53.1]	40.4 ± 4.7 (11.7)[35.2, 46.1]
Caudate nucleus**	37.6 ± 6.9 (18.4)[28.6, 49.7]	37.5 ± 6.6 (17.7)[29.8, 49.0]	− 0.1 ± 6.5[− 12.9, 12.7]	41.8 ± 5.5 (13.1)[36.1, 49.0]	33.2 ± 4.7 (14.1)[29.8, 41.8]
Putamen*	42.7 ± 6.5 (15.2)[34.5, 58.2]	43.0 ± 5.1 (11.9)[34.7, 51.2]	1.0 ± 7.1[− 12.8, 14.9]	46.8 ± 2.8 (6.0)[42.8, 51.2]	39.2 ± 3.9 (10.0)[34.7, 45.5]
Thalamus	33.6 ± 5.4 (16.0)[26.0, 43.0]	34.0 ± 4.6 (13.4)[26.1, 42.9]	1.6 ± 4.8[− 7.8, 11.1]	37.0 ± 3.6 (9.6)[32.4, 42.9]	31.0 ± 3.4 (10.9)[26.1, 35.5]
Entorhinal cortex**	47. 2± 8.6 (18.3)[35.0, 66.3]	46.4 ± 6.7 (14.5)[35.9, 55.5]	− 1.3 ± 6.9[− 14.8, 12.2]	51.1 ± 4.3 (8.4)[43.3, 55.5]	41.6 ± 5.1 (12.2)[35.9, 48.4]
Hippocampus****	52.2 ± 11.5 (22.1)[38.2, 79.2]	50.4 ± 8.9 (17.7)[38.5, 71.6]	− 2.9 ± 6.2[− 15.0, 9.2]	56.1 ± 8.3 (14.7)[49.0, 71.6]	44.7 ± 5.2 (11.6)[38.5, 51.8]
Brainstem	27.5 ± 4.2 (15.3)[22.2, 35.6]	26.9 ± 3.2 (12.0)[22.7, 32.0]	− 1.6 ± 6.4[− 14.2, 10.9]	29.1 ± 2.4 (8.2)[26.6, 32.0]	24.7 ± 2.3 (9.5)[22. 7, 28.3]

Reliability and test-retest variability of [^18^F]EKZ-001 uptake in brain were assessed for regional 2TCM and LGA *V*_*T*_ from a 120-min acquisition time interval (*n* = 4 subjects, 2M/2F, cohort B). Findings are summarized in Table 5 and indicate a higher reliability for LGA *V*_*T*_ compared to 2TCM *V*_*T*_ with an ICC consistently close to or greater than 0.80 except for the anterior cingulate (0.69) and entorhinal cortex (0.43). In terms of test-retest variability, mean TRV of 4.7 ± 11.9% and mean aTRV of 9.4 ± 8.7% were found for 2TCM *V*_*T*_ across brain regions while mean TRV and mean aTRV were 4.8 ± 7.7% and 7.7 ± 4.9%, respectively, for LGA *V*_*T*_ across brain regions. The anterior cingulate and entorhinal cortex showed the highest test-retest variability for both 2TCM and LGA *V*_*T*_, likely due to the slower kinetics in these regions.

**Table 5:** Test-retest variability assessed as $2\times \left({V}_T^{test}-{V}_T^{retest}\right)/\left({V}_T^{test}+{V}_T^{retest}\right)$ (TRV) and absolute test-retest variability assessed as $2\times \left|{V}_T^{test}-{V}_T^{retest}\right|/\left({V}_T^{test}+{V}_T^{retest}\right)$ (aTRV), averaged over the 4 test-retest datasets (*n* = 4 subjects, 2M/2F, cohort B). The intra-class correlation coefficient (ICC) is reported as measure for reliability and calculated as (BSMSS-WSMSS)/(BSMSS+WSMSS) with BSMSS and WSMSS representing the mean sum of squares between subjects and within subjects, respectively

	2TCM *V*_*T*_			LGA *V*_*T*_		
	aTRV (%)	TRV (%)	ICC	aTRV (%)	TRV (%)	ICC
Composite cortical	6.3	5.2	0.86	6.4	5.2	0.90
Orbitofrontal cortex	9.4	6.0	0.80	6.0	5.6	0.92
Frontal cortex	10.8	8.5	0.73	9.8	6.7	0.82
Motor cortex	12.1	9.9	0.64	10.1	7.3	0.78
Temporal cortex	6.8	3.9	0.87	5.7	3.7	0.90
Parietal cortex	6.0	4.2	0.88	6.6	4.7	0.89
Occipital cortex	11.2	4.5	0.78	6.1	6.1	0.93
Insula	4.9	− 0.47	0.97	5.2	3.8	0.94
Anterior cingulate	17.3	7.9	0.52	13.7	5.6	0.69
Posterior cingulate	11.0	6.6	0.71	9.1	4.4	0.84
Cerebellum	7.1	7.1	0.93	5.2	5.2	0.96
Caudate nucleus	6.5	− 3.4	0.91	5.5	5.4	0.94
Putamen	5.0	5.0	0.96	5.4	5.2	0.94
Thalamus	4.6	4.2	0.94	7.1	4.2	0.91
Entorhinal cortex	21.5	9.2	0.48	15.4	3.4	0.43
Hippocampus	13.5	− 2.3	0.79	9.5	2.2	0.80
Brainstem	5.8	4.2	0.91	3.7	3.4	0.97

**Fig. 4:**
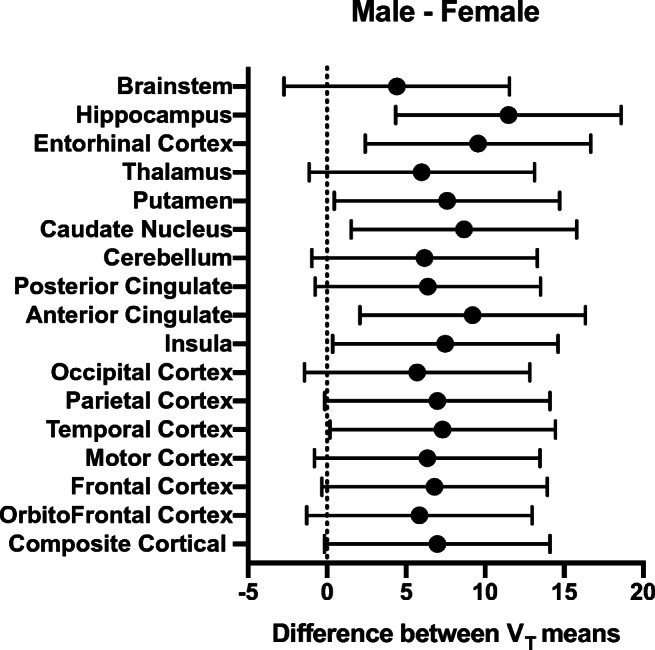
Multiple comparison plot of male and female Logan graphical analysis (LGA) *V*_*T*_ values (*n* = 6 subjects per group, cohorts B–C, only the first scan was considered for subjects with 2 scans), presenting the mean difference and 95% confidence interval of the difference

**Fig. 5:**
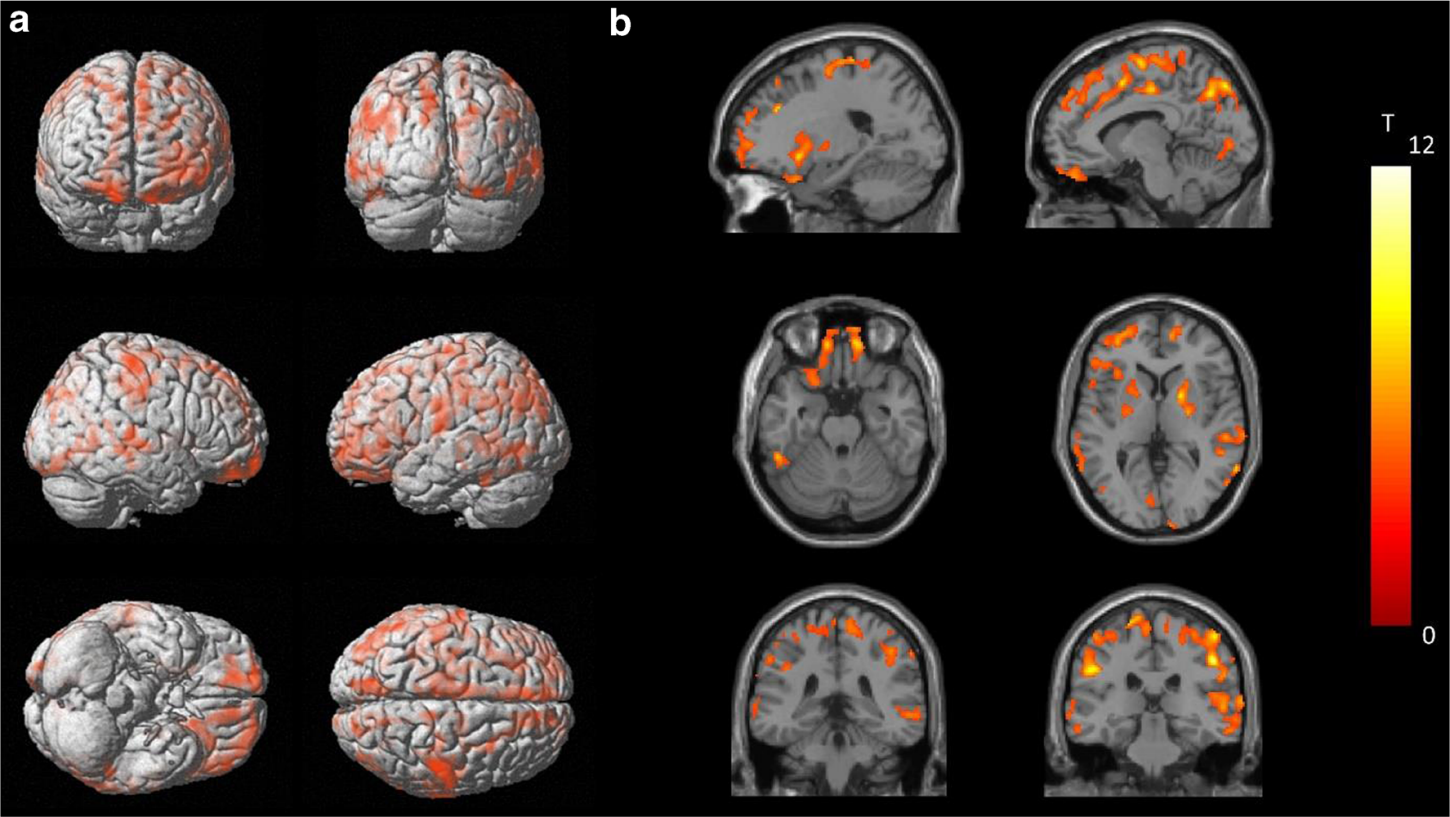
Significant clusters of increased Logan graphical analysis (LGA) *V*_*T*_ values in males compared to females (*n* = 6 subjects per group, cohorts B–C, only the first scan was considered for subjects with 2 scans) with a threshold set at *p*_height uncorr_ < 0.001 and *p*_cluster FWE-corr_ < 0.05 (no threshold kext used for the cluster extent) shown by surface rendering (**a**) and axial, coronal and sagittal slices (**b**)

Since reliability was higher and test-retest variability was lower for LGA *V*_*T*_ compared to 2TCM *V*_*T*_, we only considered LGA *V*_*T*_ for assessment of inter-subject variability and scan time reduction. Inter-subject variability (%COV) of [^18^F]EKZ-001 uptake in brain was assessed for regional LGA *V*_*T*_ from a 120-min acquisition time interval (*n* = 12 subjects, 6M/6F, cohorts B–C) and for male and female subjects separately as summarized in Table 4. Cortical inter-subject variability when considering male and female subjects separately was reduced to 7.5% and 10.6%, respectively, as compared to 13.0% for all subjects combined, indicating that inter-subject variability is partly driven by sex differences. Comparison of VOI-based LGA *V*_*T*_ values between male and female subjects (*n* = 6 per group) using a two-way ANOVA identified significant differences in the hippocampus (*p* ≤ 0.0001), anterior cingulate, caudate nucleus, entorhinal cortex (*p* ≤ 0.01), temporal cortex, insula, and putamen (*p* < 0.05) as shown in Table 4 and Fig. 4. A whole-brain voxel-wise group comparison between male and female subjects (*n* = 6 per group) demonstrated significantly higher *V*_*T*_ values (15% higher at peak level) in males, in almost all cortical and subcortical brain regions, as shown in Fig. 5a, b and Table 6.

**Table 6:** Cluster peak locations of voxel-wise group comparisons between males and females sorted according to the significance at cluster extent (*n* = 6 subjects per group, cohorts B–C, only the first scan was considered for subjects with 2 scans)

Cluster level		Voxel-level		Peak voxel coordinate			Cluster location
P_FWE-corr_	K_ext_	P_FWE-corr_	T score	*X*	*Y*	*Z*	Anatomical region
Male > female							
< 0.001	4067	0.19	9.24	− 10	− 8	66	Supplementary motor area left
		0.19	9.21	− 48	− 28	34	Supramarginal gyrus left
		0.26	8.75	− 6	− 24	66	Paracentral lobule left
		0.32	8.39	− 22	− 8	60	Superior frontal gyrus left
< 0.001	2324	0.48	7.67	− 16	40	− 16	Superior orbitofrontal gyrus left
		0.68	6.95	− 48	44	− 8	Inferior orbitofrontal gyrus left
		0.71	6.87	− 46	30	0	Inferior frontal gyrus left
		0.75	6.71	− 32	50	6	Mid frontal gyrus left
< 0.001	1712	0.15	9.67	20	48	− 14	Superior orbitofrontal gyrus right
		0.37	8.14	14	50	− 24	Superior orbitofrontal gyrus right
		0.39	8.03	12	32	34	Mid cingulum right
		0.58	7.30	34	12	60	Mid frontal gyrus right
< 0.001	1615	0.080	10.69	48	− 30	40	Supramarginal gyrus right
		0.26	8.71	50	− 28	60	Postcentral gyrus right
		0.40	7.99	10	− 32	66	Paracentral lobule right
		0.67	7.00	54	− 20	36	Postcentral gyrus right
		0.73	6.78	18	− 8	66	Superior frontal gyrus right
< 0.001	1218	0.35	8.24	− 34	12	54	Mid frontal gyrus left
		0.61	7.20	− 2	42	48	Superior frontal gyrus left
		0.62	7.16	− 46	6	16	Rolandic operculum left
		0.78	6.63	− 44	6	36	Precentral gyrus left
		0.88	6.22	− 2	22	62	Supplementary motor area left
< 0.001	901	0.26	8.73	− 44	− 74	− 4	Inferior occipital gyrus left
		0.43	7.87	− 60	− 62	− 6	Inferior temporal gyrus left
		0.59	7.28	− 52	− 42	− 22	Inferior temporal gyrus left
		0.96	5.76	− 46	− 70	− 18	Cerebellum crus 1 left
		0.96	5.76	− 42	− 60	− 8	Inferior temporal gyrus left
< 0.001	821	0.81	6.50	72	− 28	6	Superior temporal gyrus right
		0.83	6.42	62	− 32	− 6	Mid temporal gyrus right
		0.86	6.32	54	− 44	12	Mid temporal gyrus right
		0.89	6.17	70	− 18	0	Superior temporal gyrus right
		0.89	6.16	52	− 26	12	Superior temporal gyrus right
< 0.001	794	0.20	12.37	22	− 86	40	Superior occipital gyrus right
		0.41	7.94	18	− 60	18	Calcarine gyrus right
		0.63	7.13	16	− 90	30	Superior occipital gyrus right
		0.80	6.52	10	− 68	26	Cuneus right
		0.87	6.24	10	− 78	54	Superior parietal gyrus right
< 0.001	658	0.018	12.50	− 12	− 78	48	Superior parietal gyrus left
		0.22	9.00	− 8	− 66	− 42	Cerebellar 8 left
		0.82	6.45	− 12	− 88	32	Superior occipital gyrus left
		0.98	5.50	− 12	− 72	28	Cuneus left
< 0.001	529	0.23	8.94	− 18	18	− 8	Putamen left
		0.95	5.83	− 22	10	0	Putamen left
		0.99	5.29	− 26	− 8	6	Putamen left
		1.00	4.98	− 28	− 16	− 10	Hippocampus left
		1.00	4.79	− 20	16	8	Putamen left
< 0.001	425	0.21	9.10	20	6	2	Pallidum right
		0.63	7.12	26	− 8	2	Pallidum right
		0.99	5.35	26	16	2	Putamen right
0.002	320	0.16	9.57	48	− 84	− 14	Inferior occipital gyrus right
		0.25	8.78	66	− 54	2	Mid temporal gyrus right
		0.78	6.63	48	− 70	− 4	Inferior temporal gyrus right
		0.97	5.63	58	− 66	4	Mid temporal gyrus right
		0.98	5.51	50	− 60	− 6	Inferior temporal gyrus right
0.007	264	0.058	11.06	− 24	34	32	Mid frontal gyrus left
		0.65	7.06	− 28	22	38	Mid frontal gyrus left
0.008	255	0.56	7.37	28	− 84	− 16	Lingual gyrus right
		0.95	5.80	14	− 102	6	Right calcarine gyrus
0.018	216	0.91	6.08	− 14	− 82	− 2	Lingual gyrus left
		0.99	5.37	− 2	− 86	10	Calcarine gyrus left
		1.00	5.09	− 6	− 74	− 8	Lingual gyrus left
0.024	202	0.95	5.81	30	− 60	42	Angular gyrus right
		0.98	5.54	42	− 86	18	Mid occipital gyrus right
		1.00	4.79	36	− 70	38	Mid occipital gyrus right
		1.00	4.65	42	− 82	30	Mid occipital gyrus right
0.025	200	0.31	8.46	− 10	− 16	44	Mid cingulum left
0.037	181	0.62	7.17	12	− 76	− 14	Cerebellum 6 right
		0.99	5.25	16	− 62	− 10	Lingual gyrus right
		1.00	4.56	8	− 68	− 6	Lingual gyrus right
		1.00	4.46	28	− 58	− 16	Fusiform gyrus right

### Time stability—reduction of scanning time

**Fig. 6:**
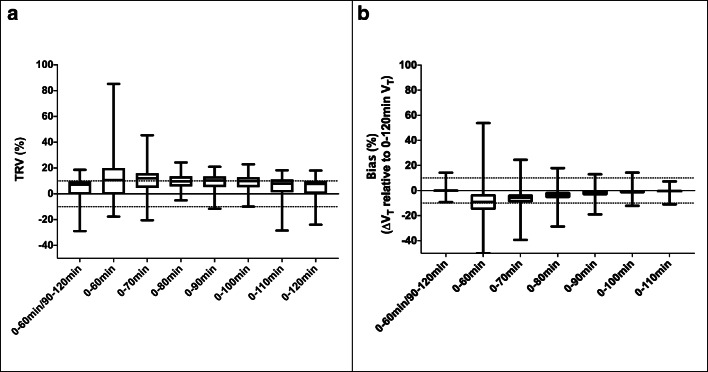
Graphical representation of the time stability of Logan graphical analysis (LGA) *V*_*T*_ for a coffee-break protocol (0–60/90–120 min) and reduced acquisition times (0–[60–120] min) compared to a 120-min dynamic acquisition protocol. Test-retest variability (TRV) defined as $2\times \left({V}_T^{test}-{V}_T^{retest}\right)/\left({V}_T^{test}+{V}_T^{retest}\right)$ for test-retest datasets (4 subjects, 2M/2F, cohort B) with dotted lines corresponding to ± 10% TRV (**a**) and bias defined as the relative difference between LGA *V*_*T*_ values using a reduced and 120-min scan time (*n* = 12 subjects, 6M/6F, cohorts B–C, only the first scan was considered for subject with 2 scans) with dotted lines corresponding to ± 10% bias (**b**)

The time stability of regional LGA *V*_*T*_ was assessed by either splitting the scan into two sessions of 60 and 30 min, respectively, with an intermediate 30-min break (coffee-break protocol) or reducing the acquisition time of one scan session down to 60 min (*n* = 12 subjects, 6M/6F, cohorts B–C). Results are presented in Table 7 and in Fig. 6a, b. Compared to a dynamic acquisition of 120 min, regional LGA *V*_*T*_ values based on the coffee-break protocol presented a very similar mean TRV of 4.5% and bias was on average smaller than 1% and within the range of − 10 to 15%. Compared to a dynamic acquisition of 120 min, regional LGA *V*_*T*_ values based on one 90-min dynamic scan session, which represents the same total acquisition time as the coffee-break protocol, presented an almost doubled mean TRV of 9.3% and bias was on average smaller than 2% and within the range of − 19 to 13%. A further reduction of the scan session to 60 min induced too high TRV and bias for regional LGA *V*_*T*_ compared to a 120-min acquisition to be considered for clinical applicability.

**Table 7:** Time stability of Logan graphical analysis (LGA) *V*_*T*_ for reduced acquisition times (0–[60–120] minutes) and a coffee-break protocol (0–60/90–120 min) evaluated as the test-retest variability (TRV) defined as $2\times \left({V}_T^{test}-{V}_T^{retest}\right)/\left({V}_T^{test}+{V}_T^{retest}\right)$ for test-retest datasets (4 subjects, 2M/2F, cohort B) and bias defined as the relative difference between LGA *V*_*T*_ values using a reduced and 120 min scan time (*n* = 12 subjects, 6M/6F, cohorts B–C, only the first scan was considered for subjects with 2 scans). Statistics are given as mean ± SD [range] and were determined across different brain regions for the subjects included

	TRV (%)	*V*_*T*_ relative to 0–120 min (%)
0–60/90–120 min	4.5 ± 7.9[− 28.9, 18.6]	0.28 ± 1.8 [− 9.3, 14.2]
0–60 min	12.1 ± 18.9[− 17.6, 85.2]	− 8.4 ± 12.6 [− 50.8, 53.8]
0–70 min	10.8 ± 10.3[− 20.5, 45.4]	− 5.9 ± 8.2[− 39.4, 24.5]
0–80 min	9.6 ± 6.3[− 5.1, 24.3]	− 3.3 ± 5.7[− 28.7, 17.9]
0–90 min	9.3 ± 6.7[− 11.6, 21.0]	− 1.6 ± 4.1 [− 19.0, 13.0]
0–100 min	8.7 ± 6.8[− 9.9, 22.9]	− 0.57 ± 3.1[− 12.2, 14.3]
0–110 min	5.9 ± 8.0[− 28.5, 18.2]	− 0.38 ± 1.5[− 11.0, 7.3]
0–120 min	4.9 ± 7.7[− 24.0, 18.0]	–

### Safety monitoring

[^18^F]EKZ-001 was safely administered with no relevant radiotracer related adverse events reported in this study. Further, no clinically relevant effects were observed in laboratory parameters, vital signs, or electrocardiogram parameters after [^18^F]EKZ-001 administration.

## Discussion

This work represents the first HDAC6 PET study in humans. We found that HDAC6 was widely expressed throughout the brain, with the highest [^18^F]EKZ-001 uptake observed in the hippocampus and entorhinal cortex, regions particularly relevant in neurodegeneration. Inhibition of HDAC6 deacetylase activity has shown beneficial effects in cell and animal models of several neurodegenerative disorders. For example, HDAC6 inhibitor treatment reduced pathological tau hyperphosphorylation and improved cognitive behaviors in mouse models of Alzheimer’s disease [14–16]. HDAC6 inhibitor treatment also increased microtubule-based intracellular transport in motor neurons and fibroblasts derived from patients with amyotrophic lateral sclerosis (ALS) [21, 22] and in *Drosophila* models of Parkinson’s disease [19] concurrent with improved motor function. Accordingly, in vivo visualization and quantification of HDAC6 using [^18^F]EKZ-001 PET across neurodegenerative disorders and along disease trajectory may uncover a role for HDAC6 in disease pathogenesis and enable stratifying the patients with the highest potential benefit from HDAC6 inhibitor treatment. Further, [^18^F]EKZ-001 PET may facilitate therapeutic development of brain penetrant HDAC6 inhibitors. Indeed, recent studies with [^18^F]EKZ-001 PET verified in vivo HDAC6 target engagement in the brain and distinguished dose-occupancy relationships between HDAC6 inhibitors in non-human primates [57].

To broadly apply [^18^F]EKZ-001 PET, robust quantitative end points are required as well as a clinically feasible acquisition protocol. Here we used full kinetic modeling and identified the 2TCM as the optimal compartment model with lower AIC values in more than 95% of cases compared to 1TCM. Differences between 2TCM and LGA *V*_*T*_ as assessed by Bland-Altman analysis were limited and support LGA as a valid non-compartment approach to generate parametric [^18^F]EKZ-001 *V*_*T*_ maps. Mean test-retest variability for both 2TCM and LGA approaches were approximately 5% across brain regions and comparable to previously published test-retest data of other PET radiotracers that require arterial blood sampling for accurate quantification [65, 66]. However, compared to 2TCM *V*_*T*_, LGA *V*_*T *_test-retest variability was generally lower, and reliability was generally higher. As LGA uses the cumulative integral of both the input function and regional TAC, LGA may be less sensitive to noise induced differences in the radiometabolite and plasma data between test and retest scanning, especially for the later sampling time points after radiotracer injection. These results therefore support the use of LGA *V*_*T*_ for [^18^F]EKZ-001 PET quantification in brain.

Interestingly, the inter-subject variability of regional LGA *V*_*T*_ across healthy adults was partly driven by sex differences. Group comparisons between males and females revealed significantly higher LGA *V*_*T*_ in males throughout cortical and subcortical regions. Notably, LGA *V*_*T*_ was 20% higher in the hippocampus and 18% higher in the motor cortex of males compared to females. Sex differences in relative aggregate HDAC expression have previously been detected throughout the limbic system with [^11^C]Martinostat [48]. Moreover, treatment with ACY-738 (an HDAC inhibitor selective for class I HDACs and HDAC6 [24, 67]) demonstrated a more beneficial effect on survival in males compared to females in an ALS mouse model [68]. Interestingly, the *HDAC6* gene is located on the X-chromosome and *HDAC6* mutation is linked to a dominant X-linked disorder in humans [69]. In sum, this illustrates that sex differences in specific HDAC paralog expression could represent a key variable of their function and should be taken into consideration for future PET studies with [^18^F]EKZ-001, which as the first paralog-selective HDAC radioligand for human use, has the unique advantage to reveal potential HDAC6 variations.

To progress [^18^F]EKZ-001 PET into diverse patient populations, reduction of scan time is necessary. We validated a coffee-break protocol that uses two separate sessions of 60 and 30 min, respectively, with an intermediate 30-min break. Regional LGA *V*_*T*_ values based on the coffee-break protocol represented similar test-retest variability and small bias compared to a 120-min dynamic acquisition. In contrast, scan time reduction to 90 min resulted in a doubled test-retest variability, whereas a further reduction to 60 min yielded a test-retest variability that was unacceptably high. Therefore, a coffee-break protocol, instead of a scan time reduction to 60 min, is preferred for patients for whom a 60-min continuous PET scan is the maximal tolerable procedure. Either a scan time reduction to 90 min or a coffee-break protocol can be considered for patients who can tolerate a 90-min continuous scan.

## Conclusion

The novel radiotracer [^18^F]EKZ-001 (also known as [^18^F]Bavarostat) is safe and has appropriate properties for clinical PET imaging of HDAC6. [^18^F]EKZ-001 biodistribution showed sufficient brain uptake and favorable dosimetry. Full kinetic modeling identified 2TCM as the most appropriate compartment model for [^18^F]EKZ-001 PET quantification in the brain, while LGA quantification provided very limited bias and better test-retest variability and reliability. Therefore, LGA *V*_*T *_is the preferred approach for [^18^F]EKZ-001 PET quantification in the brain. Males showed higher LGA *V*_*T *_values across cortical and subcortical brain regions compared to females, suggesting that sex may influence HDAC6 expression and should be considered as a variable in future studies using [^18^F]EKZ-001 PET. To increase clinical applicability, two scan sessions of 60 and 30 min, respectively, with an intermediate 30-min break can be considered instead of one dynamic 120-min scan.

## References

[1] Hubbert C, Guardiola A, Shao R, Kawaguchi Y, Ito A, Nixon A (2002). HDAC6 is a microtubule-associated deacetylase. Nature..

[2] Haggarty SJ, Koeller KM, Wong JC, Grozinger CM, Schreiber SL (2003). Domain-selective small-molecule inhibitor of histone deacetylase 6 (HDAC6)-mediated tubulin deacetylation. Proc Natl Acad Sci U S A.

[3] Cook C, Carlomagno Y, Gendron TF, Dunmore J, Scheffel K, Stetler C (2014). Acetylation of the KXGS motifs in tau is a critical determinant in modulation of tau aggregation and clearance. Hum Mol Genet.

[4] Carlomagno Y, Chung DC, Yue M, Castanedes-Casey M, Madden BJ, Dunmore J (2017). An acetylation–phosphorylation switch that regulates tau aggregation propensity and function. J Biol Chem.

[5] Kovacs JJ, Murphy PJM, Gaillard S, Zhao X, Wu JT, Nicchitta CV (2005). HDAC6 regulates Hsp90 acetylation and chaperone-dependent activation of glucocorticoid receptor. Mol Cell.

[6] Murphy PJM, Morishima Y, Kovacs JJ, Yao T-P, Pratt WB (2005). Regulation of the dynamics of hsp90 action on the glucocorticoid receptor by acetylation/deacetylation of the chaperone. J Biol Chem.

[7] Zhang X, Yuan Z, Zhang Y, Yong S, Salas-Burgos A, Koomen J (2007). HDAC6 modulates cell motility by altering the acetylation level of cortactin. Mol Cell.

[8] Dompierre JP, Godin JD, Charrin BC, Cordelières FP, King SJ, Humbert S (2007). Histone deacetylase 6 inhibition compensates for the transport deficit in Huntington’s disease by increasing tubulin acetylation. J Neurosci.

[9] Lee J-Y, Koga H, Kawaguchi Y, Tang W, Wong E, Gao Y-S (2010). HDAC6 controls autophagosome maturation essential for ubiquitin-selective qualitycontrol autophagy. EMBO J.

[10] Boyault C, Zhang Y, Fritah S, Caron C, Gilquin B, So HK (2007). HDAC6 controls major cell response pathways to cytotoxic accumulation of protein aggregates. Genes Dev.

[11] Ouyang H, Ali YO, Ravichandran M, Dong A, Qiu W, MacKenzie F (2012). Protein aggregates are recruited to aggresome by histone deacetylase 6 via unanchored ubiquitin C termini. J Biol Chem.

[12] Pandey UB, Nie Z, Batlevi Y, McCray BA, Ritson GP, Nedelsky NB (2007). HDAC6 rescues neurodegeneration and provides an essential link between autophagy and the UPS. Nature..

[13] Choi H, Kim HJ, Kim J, Kim S, Yang J, Lee W (2017). Increased acetylation of Peroxiredoxin1 by HDAC6 inhibition leads to recovery of Aβ-induced impaired axonal transport. Mol Neurodegener.

[14] Zhang L, Liu C, Wu J, Tao J-J, Sui X-L, Yao Z-G (2014). Tubastatin a/ACY-1215 improves cognition in Alzheimer’s disease transgenic mice. J Alzheimers Dis.

[15] Sung YM, Lee T, Yoon H, DiBattista AM, Song JM, Sohn Y (2013). Mercaptoacetamide-based class II HDAC inhibitor lowers Aβ levels and improves learning and memory in a mouse model of Alzheimer’s disease. Exp Neurol.

[16] Fan SJ, Huang FI, Liou JP, Yang CR (2018). The novel histone de acetylase 6 inhibitor, MPT0G211, ameliorates tau phosphorylation and cognitive deficits in an Alzheimer’s disease model article. Cell Death Dis.

[17] Govindarajan N, Rao P, Burkhardt S, Sananbenesi F, Schlüter OM, Bradke F (2013). Reducing HDAC6 ameliorates cognitive deficits in a mouse model for Alzheimer’s disease. EMBO Mol Med.

[18] Selenica ML, Benner L, Housley SB, Manchec B, Lee DC, Nash KR (2014). Histone deacetylase 6 inhibition improves memory and reduces total tau levels in a mouse model of tau deposition. Alzheimers Res Ther.

[19] Godena VK, Brookes-Hocking N, Moller A, Shaw G, Oswald M, Sancho RM (2014). Increasing microtubule acetylation rescues axonal transport and locomotor deficits caused by LRRK2 roc-COR domain mutations. Nat Commun.

[20] Jian W, Wei X, Chen L, Wang Z, Sun Y, Zhu S (2017). Inhibition of HDAC6 increases acetylation of peroxiredoxin1/2 and ameliorates 6-OHDA induced dopaminergic injury. Neurosci Lett.

[21] Guo W, Naujock M, Fumagalli L, Vandoorne T, Baatsen P, Boon R (2017). HDAC6 inhibition reverses axonal transport defects in motor neurons derived from FUS-ALS patients. Nat Commun.

[22] Heo K, Lim SM, Nahm M, Kim Y-E, Oh K-W, Park HT (2018). A de novo RAPGEF2 variant identified in a sporadic amyotrophic lateral sclerosis patient impairs microtubule stability and axonal mitochondria distribution. Exp Neurobiol.

[23] Taes I, Timmers M, Hersmus N, Bento-Abreu A, Van Den Bosch L, Van Damme P (2013). Hdac6 deletion delays disease progression in the SOD1G93A mouse model of ALS. Hum Mol Genet.

[24] Jochems J, Boulden J, Lee BG, Blendy JA, Jarpe M, Mazitschek R (2014). Antidepressant-like properties of novel HDAC6-selective inhibitors with improved brain bioavailability. Neuropsychopharmacology..

[25] Jochems J, Teegarden SL, Chen Y, Boulden J, Challis C, Ben-dor GA (2015). Enhancement of stress resilience through Hdac6-mediated regulation of glucocorticoid receptor chaperone dynamics. Biol Psychiatry.

[26] D’Ydewalle C, Krishnan J, Chiheb DM, Van Damme P, Irobi J, Kozikowski AP (2011). HDAC6 inhibitors reverse axonal loss in a mouse model of mutant HSPB1-induced Charcot-Marie-tooth disease. Nat Med.

[27] Benoy V, Van Helleputte L, Prior R, D’Ydewalle C, Haeck W, Geens N (2018). HDAC6 is a therapeutic target in mutant GARS-induced Charcot-Marie-tooth disease. Brain..

[28] Mo Z, Zhao X, Liu H, Hu Q, Chen X-Q, Pham J (2018). Aberrant GlyRS-HDAC6 interaction linked to axonal transport deficits in Charcot-Marie-tooth neuropathy. Nat Commun.

[29] Krukowski K, Ma J, Golonzhka O, Laumet GO, Gutti T, Van Duzer JH (2017). HDAC6 inhibition effectively reverses chemotherapy-induced peripheral neuropathy. Pain..

[30] Van Helleputte L, Kater M, Cook DP, Eykens C, Rossaert E, Haeck W (2018). Inhibition of histone deacetylase 6 (HDAC6) protects against vincristine induced peripheral neuropathies and inhibits tumor growth. Neurobiol Dis.

[31] Ma J, Trinh RT, Mahant ID, Peng B, Matthias P, Heijnen CJ (2019). Cell-specific role of histone deacetylase 6 in chemotherapy-induced mechanical allodynia and loss of intraepidermal nerve fibers. Pain..

[32] Ma J, Huo X, Jarpe MB, Kavelaars A, Heijnen CJ (2018). Pharmacological inhibition of HDAC6 reverses cognitive impairment and tau pathology as a result of cisplatin treatment. Acta Neuropathol Commun BioMed Central.

[33] Chiang ACA, Huo X, Kavelaars A, Heijnen CJ (2019). Chemotherapy accelerates age-related development of tauopathy and results in loss of synaptic integrity and cognitive impairment. Brain Behav Immun.

[34] Li S, Liu X, Chen X, Zhang L, Wang X (2015). Histone deacetylase 6 promotes growth of glioblastoma through inhibition of SMAD2 signaling. Tumor Biol.

[35] Wang Z, Hu P, Tang F, Lian H, Chen X, Zhang Y (2016). HDAC6 promotes cell proliferation and confers resistance to temozolomide in glioblastoma. CancerLett..

[36] Uhlen M, Oksvold P, Fagerberg L, Lundberg E, Jonasson K, Forsberg M (2010). Towards a knowledge-based human protein atlas. Nat Biotechnol.

[37] Uhlén M, Fagerberg L, Hallström BM, Lindskog C, Oksvold P, Mardinoglu A (2015). Tissue-based map of the human proteome. Science..

[38] Wey H-Y, Gilbert TM, Zürcher NR, She A, Bhanot A, Taillon BD (2016). Insights into neuroepigenetics through human histone deacetylase PET imaging. Sci Transl Med.

[39] Fukada M, Hanai A, Nakayama A, Suzuki T, Miyata N, Rodriguiz RM (2012). Loss of deacetylation activity of Hdac6 affects emotional behavior in mice. PLoS One.

[40] Odagiri S, Tanji K, Mori F, Miki Y, Kakita A, Takahashi H (2013). Brain expression level and activity of HDAC6 protein in neurodegenerative dementia. Biochem Biophys Res Commun.

[41] Ding H, Dolan PJ, Johnson GVW (2008). Histone deacetylase 6 interacts with the microtubule-associated protein tau. J Neurochem.

[42] Anderson KW, Chen J, Wang M, Mast N, Pikuleva IA, Turko IV (2015). Quantification of histone deacetylase isoforms in human frontal cortex, human retina, and mouse brain. PLoS One.

[43] Wang C, Schroeder FA, Wey HY, Borra R, Wagner FF, Reis S (2014). In vivo imaging of histone deacetylases (HDACs) in the central nervous system and major peripheral organs. J Med Chem.

[44] Schroeder FA, Wang C, Van de Bittner GC, Neelamegam R, Takakura WR, Karunakaran A (2014). PET imaging demonstrates histone deacetylase target engagement and clarifies brain penetrance of known and novel small molecule inhibitors in rat. ACS Chem Neurosci.

[45] Wey H-Y, Wang C, Schroeder FA, Logan J, Price JC, Hooker JM (2015). Kinetic analysis and quantification of [11C] Martinostat for in vivo HDAC imaging of the brain. ACS Chem Neurosci.

[46] Donovan LL, Magnussen JH, Dyssegaard A, Lehel S, Hooker JM, Knudsen GM (2020). Imaging HDACs in vivo: cross-validation of the [11C] Martinostat radioligand in the pig brain. Mol Imaging Biol.

[47] Gilbert TM, Zürcher NR, Wu CJ, Bhanot A, Hightower BG, Kim M (2018). PET neuroimaging reveals histone deacetylase dysregulation in schizophrenia. J Clin Invest.

[48] Gilbert TM, Zürcher NR, Catanese MC, Tseng CEJ, Di Biase MA, Lyall AE (2019). Neuroepigenetic signatures of age and sex in the living human brain. Nat Commun.

[49] Dios AM, Babu S, Granucci EJ, Mueller KA, Mills AN, Alshikho MJ (2019). Class I and II histone deacetylase expression is not altered in human amyotrophic lateral sclerosis: Neuropathological and positron emission tomography molecular neuroimaging evidence. Muscle Nerve.

[50] Pigna E, Simonazzi E, Sanna K, Bernadzki KM, Proszynski T, Heil C (2019). Histone deacetylase 4 protects from denervation and skeletal muscle atrophy in a murine model of amyotrophic lateral sclerosis. EBioMedicine..

[51] Chen S, Zhang X-J, Li L-X, Wang Y, Zhong R-J, Le W (2015). Histone deacetylase 6 delays motor neuron degeneration by ameliorating the autophagic flux defect in a transgenic mouse model of amyotrophic lateral sclerosis. Neurosci Bull.

[52] Janssen C, Schmalbach S, Boeselt S, Sarlette A, Dengler R, Petri S (2010). Differential histone deacetylase mRNA expression patterns in amyotrophic lateral sclerosis. J Neuropathol Exp Neurol.

[53] Lee J-Y, Kawaguchi Y, Li M, Kapur M, Choi SJ, Kim H-J (2015). Uncoupling of protein aggregation and neurodegeneration in a mouse amyotrophic lateral sclerosis model. Neurodegener Dis.

[54] Lu S, Zhang Y, Kalin JH, Cai L, Kozikowski AP, Pike VW (2016). Exploration of the labeling of [11C] tubastatin a at the hydroxamic acid site with [11C] carbon monoxide. J Label Compd Radiopharm.

[55] Strebl MG, Campbell AJ, Zhao WN, Schroeder FA, Riley MM, Chindavong PS (2017). HDAC6 brain mapping with [18F] Bavarostat enabled by a Ru-mediated deoxyfluorination. ACS Cent Sci.

[56] Vermeulen K, Ahamed M, Luyten K, Bormans G (2019). Evaluation of [11C]KB631 as a PET tracer for in vivo visualisation of HDAC6 in B16.F10 melanoma. Nucl Med Biol.

[57] Celen S, Rokka J, Gilbert TM, Koole M, Vermeulen I, Serdons K (2020). Translation of HDAC6 PET imaging using [18F]EKZ-001–cGMP production and measurement of HDAC6 target occupancy in nonhuman primates. ACS Chem Neurosci.

[58] ICRP (1979). Publication 30, part 1, 1979. Ann ICRP.

[59] Stabin MG, Sparks RB, Crowe E (2005). OLINDA/EXM: the second-generation personal computer software for internal dose assessment in nuclear medicine. J Nucl Med.

[60] Johansson L, Mattsson S, Nosslin B, Leide-Svegborn S (1992). Effective dose from radiopharmaceuticals. Eur J Nucl Med.

[61] Schramm G, Koole M, Willekens SMA, Rezaei A, Van Weehaeghe D, Delso G (2019). Regional accuracy of ZTE-based attenuation correction in static [F]FDG and dynamic [F]PE2I brain PET/MR. Front Phys.

[62] Khalifé M, Fernandez B, Jaubert O, Soussan M, Brulon V, Buvat I (2017). Subject-specific bone attenuation correction for brain PET/MR: can ZTE-MRI substitute CT scan accurately?. Phys Med Biol.

[63] Logan J, Fowler JS, Volkow ND, Wolf AP, Dewey SL, Schlyer DJ (1990). Graphical analysis of reversible radioligand binding from time-activity measurements applied to [N-11C-methyl]-(−)-cocaine PET studies in human subjects. J Cereb Blood Flow Metab.

[64] Zanotti-Fregonara P, Lammertsma AA, Innis RB (2013). Suggested pathway to assess radiation safety of 18F-labeled PET tracers for first-in-human studies. Eur J Nucl Med Mol Imaging.

[65] Arakawa R, Takano A, Stenkrona P, Stepanov V, Nag S, Jahan M, et al. PET imaging of beta-secretase 1 in the human brain: radiation dosimetry, quantification, and test-retest examination of [18F]PF-06684511. Eur J Nucl Med Mol Imaging. 2020:1–11. 10.1007/s00259-020-04739-5. 10.1007/s00259-020-04739-5 PMC7396399 32140803

[66] Van Weehaeghe D, Koole M, Schmidt ME, Deman S, Jacobs AH, Souche E (2019). [11C]JNJ54173717, a novel P2X7 receptor radioligand as marker for neuroinflammation: human biodistribution, dosimetry, brain kinetic modelling and quantification of brain P2X7 receptors in patients with Parkinson’s disease and healthy volunteers. Eur J Nucl Med Mol Imaging.

[67] Mithraprabhu S, Khong T, Jones SS, Spencer A (2013). Histone deacetylase (HDAC) inhibitors as single agents induce multiple myeloma cell death principally through the inhibition of class I. HDAC.

[68] Rossaert E, Pollari E, Jaspers T, Van Helleputte L, Jarpe M, Van Damme P (2019). Restoration of histone acetylation ameliorates disease and metabolic abnormalities in a FUS mouse model. Acta Neuropathol Commun BioMed Central.

[69] Simon D, Laloo B, Barillot M, Barnetche T, Blanchard C, Rooryck C (2010). A mutation in the 3′-UTR of the HDAC6 gene abolishing the post-transcriptional regulation mediated by hsa-miR-433 is linked to a new form of dominant X-linked chondrodysplasia. Hum Mol Genet.

